# From Diagnosis to Disclosure: Navigating Genetic Counseling Dilemmas in *BRCA2*‐Associated Prostate Cancer in the Middle East

**DOI:** 10.1155/crig/2823337

**Published:** 2026-07-22

**Authors:** Chantel van Wyk, Reem Abdulrahim, Ilse Crous, Abeer Al Saegh, Amira Al Balushi, Bushra Al Muhairi, Sara Al Kiyumi, Suad Al Malki, Munjid Al Harthy

**Affiliations:** ^1^ Genomics Department, Sultan Qaboos Comprehensive Cancer Care and Research Center (SQCCCRC), Muscat, Oman; ^2^ Genetics and Genomics Department, College of Medicine and Health Sciences, United Arab Emirates University, Al Ain, Abu Dhabi, UAE, uaeu.ac.ae; ^3^ Genitourinary Programme, Sultan Qaboos Comprehensive Cancer Care and Research Center (SQCCCRC), Muscat, Oman

**Keywords:** *BRCA2* cascade testing, collectivist societies, cultural norms, family communication, genetic counseling, prostate cancer

## Abstract

**Background:**

Germline genetic testing is integral to precision oncology in prostate cancer (PCa), particularly for men with pathogenic or likely pathogenic (P/LP) *BRCA1/2* variants, where results have therapeutic and hereditary implications. However, data from the Middle East remain limited regarding real‐world implementation of genetic counseling and its influence on family communication and cascade testing.

**Aim:**

To describe the role and challenges of genetic counseling for *BRCA2*‐associated PCa in Omani patients, focusing on family communication, risk awareness, and cascade testing uptake.

**Methods:**

This descriptive, retrospective case series included nine men with PCa carrying P/LP *BRCA2* variants identified between September 2021 and December 2025. Clinical and genetic data were extracted from electronic records, and genetic counseling documentation was reviewed for family history, psychosocial context, communication patterns, and cascade testing. Quantitative data were summarized descriptively. Qualitative content analysis of counseling records identified recurring sociocultural and communication‐related factors.

**Findings and Discussion:**

Patients were aged 60–76 years at the time of PCa diagnosis, with varied educational backgrounds and disease stages, and all carried *BRCA2* variants. Awareness of diagnosis differed, with some patients partially informed or unaware. These findings underscore the pivotal role of genetic counseling in translating *BRCA2* molecular results into actionable strategies for patients and families. Family‐mediated protective nondisclosure and decision‐making were prominent. Cascade testing uptake was higher in families with younger or medically informed relatives and lower where stigma or fear prevailed.

**Conclusion:**

In this single‐center Omani case series, effective counseling for *BRCA2*‐associated PCa required navigating complex cultural dynamics related to family‐mediated communication and decision‐making. Integrating culturally sensitive, family‐centered genetic counseling into routine oncology care may save lives by empowering families to act earlier on inherited risk.

## 1. Introduction

Prostate cancer (PCa) is one of the most common malignancies in men worldwide [1]. In Oman, it is a leading cause of cancer diagnosis in men, with rising incidence rates in the past few years [2, 3]. Another well‐documented characteristic of PCa in Oman is that a higher proportion of Omani men are diagnosed with advanced or metastatic disease than in Western populations [3, 4], underscoring the need for improved risk stratification, preventive strategies, and public awareness [5].

Germline testing for breast cancer genes 1 and 2 (*BRCA1/2)* and other homologous recombination repair (HRR) genes has become an essential component of PCa management [6]. Pathogenic or likely pathogenic (P/LP) variants in *BRCA1/2* are associated with aggressive disease and have important therapeutic, prognostic, and familial implications [7–9]. Approximately 15% of men with advanced PCa can have P/LP germline variants [6]. The current National Comprehensive Cancer Network (NCCN) guidelines, adopted in the Middle East and North Africa (MENA) region, recommend germline testing in men with metastatic or high‐risk PCa and those with a significant family history of breast, ovarian, pancreatic, or PCa [6, 8]. At the Sultan Qaboos Comprehensive Cancer Care and Research Center (SQCCCRC), testing typically begins with *BRCA1/2* sequencing, followed by a comprehensive cancer panel that evaluates 154 genes.

In addition to guiding therapy, germline testing helps identify at‐risk relatives through cascade testing. Genetic counseling plays a crucial role in obtaining family history, facilitating informed consent, understanding clinical issues, and addressing psychosocial issues related to risk communication within families [6, 10].

However, engagement of men with genetic counseling is still inconsistent. Studies outside of the Middle East indicate that men may underestimate the risk of inherited cancers or view *BRCA*‐related cancer as a women‐related disease [11–14]. There is a lack of information from the MENA region, especially regarding the implementation of genetic counseling as a means of family communication and a tool to improve the uptake of cascade testing [8, 15]. In Oman, cancer genetic counseling services were first initiated at two different tertiary care hospitals in 2012 and then introduced at SQCCCRC in 2021. Although facilities for testing and genetic counseling are under development, the practical application of cascade testing and family risk communication remains underreported [16].

The Middle Eastern context presents some unique factors. In collectivist cultures such as Oman, health‐related decision‐making can be strongly influenced by family dynamics. The disclosure of cancer and genetic information can be negotiated collectively, and family‐mediated protective nondisclosure may be practiced to protect family members from distress [17, 18]. The prevalence of consanguineous marriages, strong family hierarchies, gendered views on cancer risk, and communication styles that value privacy or concealment can also shape how genetic risk is understood and shared [16, 19]. In these settings, autonomy can be collective rather than individual, and culturally informed counseling practices may be needed [20].

The objective of this case series is to explore the role and challenges of genetic counseling in men with *BRCA2*‐related PCa in Oman, with particular attention to family and sociocultural influences on risk communication and variability in cascade testing uptake. By examining counseling processes across diverse families, this study seeks to deepen understanding of how genetic counseling is practiced in the Omani context and to inform the development of client‐centered, culturally responsive approaches to hereditary cancer care.

## 2. Methodology

### 2.1. Study Design, Setting, and Patient Selection

This retrospective case series investigates the genetic counseling pathways of Omani men diagnosed with PCa at the SQCCCRC between September 2021 and December 2025. The reporting of this study was guided by the Case Report (CARE) guidelines for clinical case series. Patients were identified through the institutional genomics database. During the study period, a total of 135 men with PCa underwent cancer genetic counseling and 130 underwent germline genetic testing at our institution. All probands received standard pretest genetic counseling addressing hereditary cancer risk, possible genetic test outcomes (P/LP variant, variant of uncertain significance [VUS], negative result, or incidental finding), clinical implications, and psychosocial considerations. Post‐test counseling focused on result disclosure and recommendations for cascade testing among at‐risk relatives. Predictive genetic counseling and cascade testing were provided to at‐risk relatives after the test results were disclosed.

For this specific case series, inclusion criteria were restricted to men with PCa who tested positive for a P/LP variant in *BRCA1 or BRCA2*. Patients were excluded if they tested negative, carried only VUSs, or had pathogenic variants in non‐*BRCA* genes. Based on these criteria, nine men were purposefully selected for inclusion to explore the unique counseling dynamics associated with *BRCA*‐associated PCa in our clinical setting. None of the men had a *BRCA1* P/LP variant identified. These nine cases constitute all *BRCA2*‐positive PCa probands evaluated at the center during the study period; no eligible cases were excluded.

### 2.2. Genetic Testing Strategy and Variant Classification

As the cancer center was still establishing its internal genomics laboratory at the time of this study, early genetic testing was initiated using an in‐house *BRCA1/2* test utilizing Sanger sequencing. However, this in‐house assay did not include multiplex ligation–dependent probe amplification (MLPA) for deletion/duplication analysis. To ensure comprehensive evaluation and to verify the in‐house findings, patient samples were subsequently sent for external testing at an accredited facility (Fulgent Laboratories, USA). The external testing utilized a comprehensive multigene cancer panel covering up to 154 genes. This panel included full‐focus genes associated with hereditary cancer syndromes, as well as candidate genes with newly discovered cancer associations (where risks may currently be unclear but could become actionable as new evidence emerges), and complete deletion/duplication analysis. Variants were classified according to the American College of Medical Genetics and Genomics and the Association for Molecular Pathology (ACMG/AMP) standards, and P/LP variants and variants of unknown significance were reported. For the purpose of this analysis, variant classifications were cross‐referenced with the ClinVar database (last checked in December 2025). Six patients had in‐house *BRCA1/2* sequencing followed by a comprehensive cancer panel, but in three cases (Cases 2, 4, and 6), targeted variant analysis was performed in‐house as the PV was first identified in a family member who was cared for at SQCCCRC.

### 2.3. Data Collection and Qualitative Analysis

Quantitative clinical and demographic data (including age at diagnosis, education, disease stage, and initial PSA), alongside genetic test results, were extracted from electronic patient records and summarized descriptively to characterize the cohort and document cascade testing uptake. Concurrently, pre‐ and post‐test genetic counseling documentation was systematically reviewed to extract qualitative data regarding family history, psychosocial context, and intrafamily communication patterns.

To ensure analytical rigor, a structured qualitative content analysis was conducted. Data extraction and coding were performed independently by two researchers (C.V.W. and R.A.). An initial codebook was developed iteratively based on a preliminary review of three cases, focusing on themes such as “family‐mediated protective nondisclosure,” “gendered risk perception,” and “barriers to cascade testing.” The remaining cases were coded using this framework. Any discrepancies in coding or thematic categorization between the two independent reviewers were resolved through consensus discussion, with a third reviewer (I.C.) consulted when necessary. For each proband, the counseling pathway was mapped chronologically, from referral to pretest counseling, test result disclosure, and subsequent cascade counseling, to identify key decision points and communication patterns. Narrative case summaries were developed to illustrate representative counseling scenarios within the cohort.

### 2.4. Ethical Considerations

Ethical approval was obtained from the SQCCCRC Institutional Review Board and Ethics Committee (SQCCCRC‐IRB&EC/160/2024/CCCRC‐76‐2024). To ensure confidentiality within a small national context, all data were anonymized using unique study codes, and specific identifying family structures were obscured in the published vignettes in accordance with institutional privacy policies. However, specific clinical combinations (such as rare cancer types, ages at diagnosis, and variants) were retained, as they were explicitly approved for publication through the informed consent process and are strictly necessary for the scientific interpretation of the cases. Written informed consent explicitly covering the clinical procedure, the use of anonymized clinical data for research, and the publication of detailed case narratives was obtained for all cases. Because of the varying degrees of family‐mediated disclosure in this cohort, the consent framework was managed individually according to patient and/or family preferences and institutional policy:

• Standard Direct Consent (Cases 2, 4, 6, 8, and 9): The proband was fully counseled, aware of their cancer diagnosis, and directly provided written informed consent for the genetic testing, research participation, and publication of their case narrative.
• Delegated Result Disclosure (Cases 1 and 7): The probands signed the initial consent forms for genetic testing and research themselves. However, during pretest counseling, they formally delegated the receipt of results and subsequent healthcare decision‐making to their adult children. In these instances, the designated surrogate family members also provided supplementary consent for the publication of the family’s counseling narratives.
• Delegated Testing with Shared Disclosure (Case 5): The proband initially delegated the decision to undergo genetic testing to his children. However, following the testing process, he was included, alongside his children, in the result disclosure consultation and provided direct consent for the publication of his clinical journey.
• Full Surrogate Consent (Case 3): The proband was excluded from disclosure regarding both his cancer diagnosis and genetic status due to family‐mediated protective nondisclosure. In this case, surrogate written consent was obtained from the primary next of kin for both clinical testing and the publication of case details, in strict accordance with institutional ethics policies regarding family‐mediated health care.

## 3. Series Cohort Overview and Findings

### 3.1. Cohort Overview

This series describes nine Omani men with PCa and germline *BRCA2* variants (P/LP) who attended genetic counseling. The age range at the time of PCa diagnosis varied from 60 to 76 years, with most men being retired and having educational attainment ranging from no formal schooling to postgraduate education. Seven of the nine men had metastatic or high‐risk disease, and two had multiple other primary cancers (male breast cancer or colorectal cancer). The demographic, clinical, and genetic features of the probands are listed in Table 1. The family history ranged from putatively unaffected to highly suggestive of hereditary cancer. A family history of colon cancer (with multiple first‐ or second‐degree relatives affected) was present in three of the nine men.

**TABLE 1:** Demographic, clinical, and genetic characteristics of the proband cohort (*n* = 9).

Case	Age at consult	Age at PCa Dx	Education	Employment	PCa presentation	Metastatic sites	Other primary malignancies	iPSA (ng/mL)	Gleason score	Family history of *BRCA*‐associated cancers	*BRCA2* variant
1	76	74	None	Unemployed	De novo metastatic CRPC	Bone, LN	None	820	10 (5 + 5)	Breast (first and second degrees)	Pathogenic ^a
2	68	60	Primary	Retired	CRPC; local recurrence	None	None	162	8 (4 + 4)	Breast (first–third degrees), PCa (first degree)	Pathogenic ^b
3	79	76	University	Retired	Oligometastatic hormone‐sensitive	Bone, LN	None	62	NR	Breast (second degree)	Pathogenic ^c
4	69	63	Primary	Employed	Localized high risk	None	Male breast (DCIS)	NR	NR	Breast (first and second degrees)	Pathogenic ^d
5	71	63	None	Unemployed	Metastatic CRPC	Lungs, bone, LN	None	24.6	7 (4 + 3)	PCa (first degree)	Pathogenic ^d
6	63	60	Primary	Retired	Metastatic	Retroperitoneal LN	Bladder	NR	10 (5 + 5)	Breast (first–third degrees)	Pathogenic ^e
7	74	74	None	Self‐employed	Metastatic, high volume	Bone, LN	None	838	NR	None	Pathogenic ^d
8	71	65	University	Retired	Localized	None	Colorectal	NR	NR	PCa and breast (first and second degrees)	Likely pathogenic ^f
9	67	66	Primary	Employed	High‐risk localized	None	None	10.5	10 (5 + 5)	None	Pathogenic ^g

*Note:* Although VUS is not listed in this specific table, variants of uncertain significance were identified in the cohort and are discussed in the text). ^a c.3195 + 3198del (p.Asn1066Leufs*10)^b c.9382C > T (p.*Arg*3128*) ^c c.6302dup ^d c.2588dup (p.Asn863Lysfs)*^e deletion of exon 3^f c.1394_1403del (p.Val465Glufs*17) ^g c.5290_5291del (p.Ser1764Lysfs∗3). Dx, diagnosis; VUS, variant of uncertain significance; PCa, prostate cancer.

Abbreviations: CRPC, castration‐resistant prostate cancer; DCIS, ductal carcinoma in situ; iPSA, initial prostate‐specific antigen; LN, lymph nodes; NR, not recorded; PSA, prostate‐specific antigen.

### 3.2. Impact on Precision Oncology and High‐Risk Screening

Although the primary focus of this study is on genetic counseling dynamics, the identification of pathogenic *BRCA2* variants also has direct clinical implications for precision oncology and surveillance. Two patients (Cases 1 and 5) were initiated on targeted therapy with PARP inhibitors (e.g., olaparib), though both subsequently required alternative management due to tolerability issues and adverse side effects; ultimately, one of these patients passed away before further therapeutic decisions could be made. For patients who were already demonstrating undetectable PSA levels or optimal responses to their current therapies (such as apalutamide or abiraterone in Cases 2, 3, 6, and 7), existing treatment regimens were maintained. Furthermore, the *BRCA2* results guided tailored high‐risk screening protocols for several patients in remission or with controlled disease, integrating regular surveillance for male breast cancer, pancreatic cancer, and melanoma into their routine care (Cases 4, 7, and 8). In one instance (Case 9), advanced disease and comorbid psychiatric conditions impeded the execution of an expanded screening plan.

The genetic counseling pathways, family communication, and cascade testing outcomes are shown in Table 2. There were three patterns observed in the cohort:

1. Variable patient awareness of cancer diagnosis and prognosis, influenced by family‐mediated protective nondisclosure
2. The profound impact of cultural norms, including collectivism (decision‐making, family communication patterns)
3. Heterogeneous adoption of cascade testing

**TABLE 2:** Genetic counseling pathways, family communication, and cascade testing outcomes.

Case	The patient is aware of their PCa Dx	Genetic result disclosed to the proband	Primary counseling recipients	Family‐mediated protective nondisclosure	Number of children^∗^	Number of siblings^∗^	At‐risk relatives tested^∗∗^
1	Partial	No	Adult children	Yes	16–20	1–5	High (multiple generations)
2	Yes	Yes	Proband and family	No	1–5	1–5	Low
3	No	No	Wife and adult children	Yes	6–10	6–10	High
4	Yes	Yes	Proband and relatives	Partial	6–10	1–5	Moderate
5	Partial	Yes	Proband	Yes	6–10	6–10	Low
6	Yes	Yes	Proband and family	Yes	6–10	11–15	Low
7	Partial	No	Adult children	Yes	16–20	0	High
8	Yes	Yes	Proband	Partial	1–5	11–15	Low
9	Yes	No	Proband and niece	Yes	6–10	1–5	Moderate

Abbreviations: Dx, diagnosis; PCa, prostate cancer.

^∗^To minimize the risk of participant identification in a small national context, the number of children and siblings eligible for predictive genetic counseling and testing is reported as aggregated ranges.

^∗∗^Cascade testing uptake was categorized based on documented counseling and testing at the last follow‐up: high = ≥ 50% of identified first‐degree relatives tested; moderate => 1 but < 50% tested; low = no relatives or only 1 relative tested.

The following sections will present cases as illustrative vignettes to highlight the variety of clinical presentations, family structures, and sociocultural factors, as well as their implications for genetic counseling and cascade testing in the Omani setting.

### 3.3. Case Summaries

#### 3.3.1. Case 1

The proband was a 76‐year‐old man with no formal education, who was diagnosed at the age of 74 with de novo metastatic high‐volume PCa (initial PSA of 820 ng/mL, Gleason score of 5 + 5 = 10). At the time of referral to the genetic clinic, the proband was aware of his cancer diagnosis but did not know of his prognosis. He underwent pretest genetic counseling and gave consent for germline testing, which included the option of sharing the results with his adult children in his absence. The patient was placed on PARP inhibitors, but this was discontinued due to tolerability; the patient’s disease progressed, and he died within a few months after his diagnosis.

The family history showed several cases of malignancy in first‐ and second‐degree relatives, including prostate, breast, and colorectal cancers, in a consanguineous family structure. Two of the proband’s children had died of breast cancer in their early 40s and colorectal cancer in their early 40s, and a third was diagnosed with breast cancer in their late 30s after the proband’s genetic test disclosure. Other malignancies were also present in siblings, nieces, and nephews. Previous genetic analysis performed in 2015 at another tertiary institution identified a pathogenic *BRCA2* variant in affected maternal female cousins with breast cancer. This information was not known at the time of the proband’s referral. Moreover, two *PMS1* variants, homozygous c.1383del (p.Val462Leufs14) and heterozygous c.1706G > A (p.Arg569Gln), were found in a maternal male cousin with colon cancer. The c.1383del variant was initially classified as pathogenic but was subsequently reclassified as a VUS, a classification it retains to date. In contrast, the c.1706G > A variant has consistently been classified as a VUS. This information was known as this patient was also a genetic counseling patient at SQCCCRC. He did not have the *BRCA2* PV.

In our proband, comprehensive germline testing revealed a pathogenic *BRCA2* variant consistent with a previously known familial mutation, as well as five variants of unknown significance. There was no *PMS1* variant in the proband. However, the result of the genetic test, which was also discovered later, in his late son with colon cancer, revealed the presence of the c.1383del *PMS1* variant, which most likely originated from the maternal lineage. The proband was married to his first cousin.

At the time of disclosing the result, the patient was not doing well clinically and psychologically, as reported by his family members. The children decided collectively not to disclose the pathogenic genetic test result to their father. The reason for this decision was that they did not want to distress him further, so he could focus on his own health and spare him worry about his offspring. Although the proband initially agreed to the disclosure of the result to his children, it was still difficult for the counselor to exclude him from the discussion.

Cascade counseling was extended to at‐risk relatives, resulting in predictive counseling and genetic testing uptake among the majority of the proband’s children (two‐thirds) and several additional family members. Testing identified the familial *BRCA2* pathogenic variant in an adult female relative who was later diagnosed with breast cancer.

#### 3.3.2. Case 2

The proband was a 68‐year‐old retired man with primary‐level education, diagnosed at the age of 60 with castration‐resistant PCa (initial PSA level of 162 ng/mL, Gleason score 4 + 4 = 8). He also experienced a localized recurrence after definitive treatment. The patient demonstrated good understanding of his condition and regularly attended appointments with the oncologist and genetic counselor, sometimes accompanied by his wife and eldest son.

Family history was significant for early‐onset and recurrent breast cancer in his daughter, who was diagnosed in her late 20s with contralateral disease in her 40s, and PCa in his brother. Further breast cancer diagnoses were reported in nieces and maternal cousins. A pathogenic *BRCA2* variant was found in the proband’s daughter in 2015. The patient was unable to recall whether he had undergone genetic testing, and no records could be found. However, the patient did say that he had had follow‐up for regular screening because of his daughter’s breast cancer. To our knowledge, no other family members had had predictive genetic counseling and testing, nor had they joined formal high‐risk screening programs because of his daughter’s first cancer diagnosis. There was also evident variation in knowledge of hereditary cancer risk within branches of this family because communication about cancer and genetic risk outside of the close familial relationship was not practiced.

Targeted genetic testing has confirmed that the proband inherited a pathogenic *BRCA2* variant. During genetic counseling, the patient stated that he discussed the hereditary risk of cancer with his children and siblings. Despite repeated genetic counseling and follow‐up since the daughter’s test in 2015, none of the proband’s young‐adult children have undergone predictive genetic testing as of this report. This includes the patient’s eldest son, who has attended all the current genetic counseling sessions with his father. Some of the practical reasons that were given for not coming forward for predictive genetic testing by the first‐degree relatives include that male relatives felt that genetic testing is more relevant to women because of the association with breast cancer, and the younger ones had study and work commitments that made it difficult to come in for genetic counseling and testing. The proband’s wife also stated that there was some concern about the emotional implications of the test result on the children.

#### 3.3.3. Case 3

The proband was a 79‐year‐old retired man with university‐level education, diagnosed three years earlier, at 76 years old, with oligometastatic hormone‐sensitive PCa (initial PSA of 62, Gleason score not recorded). Despite his regular engagement with healthcare services within a specialized cancer center, the patient was reportedly not aware of his cancer diagnosis, following a family decision to keep this information from him.

Family history included several instances of breast cancer on both the maternal and paternal sides, including two nieces diagnosed in their 50s. The proband was married to a distant relative, and the family pedigree was notable for widespread consanguinity, including multiple consanguineous unions among extended family members. There were also reports of two distant relatives with another autosomal recessive genetic disorder. Comprehensive germline testing identified a pathogenic *BRCA2* variant, along with two additional pathogenic germline findings that were not considered relevant to the present report. The findings showed a complex pattern of inherited cancer risk, with cumulative effects for various risks of malignancy, particularly for at‐risk relatives in this family with several consanguineous marriages.

Pre‐ and post‐test counseling was primarily conducted with family members. The counselor faced the difficult task of balancing cultural values and promoting patient autonomy. Ethical issues were raised, especially regarding informed consent and patient rights. Nevertheless, the relatives chose not to reveal the patient’s malignancy and genetic status.

Based on the result disclosure, predictive counseling and testing were conducted on the proband’s wife, almost all of his children, and all children of the proband’s affected sister. Additional testing of maternal relatives was suggested and remained pending at the time of reporting.

#### 3.3.4. Case 4

The proband was a 69‐year‐old man who was diagnosed with PCa (details of the disease were not recorded) at the age of 63 and later referred for genetic testing after being diagnosed with left‐sided high‐grade ductal carcinoma in situ of the breast at the age of 69. The proband was fully informed about both conditions and showed good health literacy and participation with the genetic counselor.

Family history included both colon cancer diagnosis (late 30s) and breast cancer in a sister (50s), breast cancer in a niece (early 30s), and colon cancer in the father (50s). The clustering of early‐onset cancers and multiple primary malignancies in first‐degree relatives was highly suggestive of an inherited predisposition. Germline testing, which initially took place in the sister, also treated at SQCCCRC, revealed a pathogenic *BRCA2* variant (c.2588dup). The family was very open about their discussion of cancer within their family and reported that they had informed all the close relatives and provided them with contact information about the genetics clinic. Despite this, the proband did not present for genetic evaluation until his own referral for genetic testing after being diagnosed with male ductal carcinoma in situ of the breast. The *BRCA2* variant was later confirmed in the proband through targeted variant analysis.

Predictive counseling and testing were made available to the proband’s children. Some of the proband’s siblings, nieces, and nephews sought testing. Interestingly, none of the proband’s children (some of whom were young and still unmarried) had undergone cascade testing at the time of reporting. The proband reported that his children felt emotionally unprepared to accept the potential outcome.

#### 3.3.5. Case 5

The proband was a 71‐year‐old retired man with no formal education, diagnosed with high‐risk PCa at the age of 63 (initial PSA 24.6 ng/mL, Gleason score 4 + 3 = 7), with recent disease progression. At the time of referral, he was aware of his initial diagnosis, but not of the disease progression. His low literacy level may have influenced his perception of his diagnosis and his interaction with the healthcare system. He came to the genetic consultation alone.

Family history was restricted to a single brother who died of PCa in his early 60s. The construction of the extended pedigree was difficult due to a lack of documentation and the scattering of family members, as most of his relatives had migrated to Africa.

Germline analysis revealed a pathogenic *BRCA2* variant (c.2588dup) and three VUSs. The same *BRCA2* variant and surname were shared with another proband in this series. Mapping their backgrounds, they shared a similar regional origin, suggesting possible clustering by region.

The uptake of cascade testing remained low, with no family members having undergone predictive counseling and cascade testing at the time of reporting. After the disclosure of the test results, efforts were made to contact relatives for potential referrals to predictive counseling; however, communication barriers, low health literacy, and unclear relationships with extended relatives hindered immediate uptake of cascade testing.

#### 3.3.6. Case 6

The proband was a 63‐year‐old retired man with primary education, who was diagnosed with metastatic PCa (initial PSA not recorded, Gleason score of 5 + 5 = 10) three years prior, at 60 years old, in this study, and with bladder cancer one year later. He demonstrated awareness of both conditions and participated in follow‐up care and genetic counseling.

Family history included a daughter who was diagnosed with pregnancy‐associated breast cancer in her late 20s, and she was found to have a *BRCA2* pathogenic variant. The proband has more than 10 siblings; one sister was diagnosed with breast cancer in her early 30s. On the proband’s maternal side, there was a multigenerational family history of breast cancer, where an aunt and her daughter were both diagnosed with breast cancer in their 40s–50s. There were also four daughters of another asymptomatic maternal aunt who were diagnosed with breast cancer in their 40s. The extended family members received medical treatment and genetic testing at another hospital in the region. However, information regarding the family’s cancer history and genetic test results was not communicated within the family. As a result, neither the proband nor his daughter was aware of the family’s hereditary cancer risk until they were diagnosed with cancer. Consequently, the proband did not undergo formal high‐risk cancer screening. At the time of referral, we were able to connect the proband’s medical file with his daughter’s file and obtain additional information regarding the family history and previously reported genetic test results.

Targeted testing confirmed the presence of a pathogenic *BRCA2* variant in the proband. Despite the number of counseling sessions conducted to educate the patient and his family members on the importance of cascade testing and early screening, the proband’s adult children had not undergone testing at the time of reporting. It is possible that the children were still unaware of their siblings’ cancer diagnosis and family history.

#### 3.3.7. Case 7

The proband was a 74‐year‐old man diagnosed with metastatic high‐volume PCa (initial PSA of 838 ng/mL). The proband denied any known family history of malignancy. The proband had more than 15 young‐to‐middle‐aged children. The family members censored the medical and health information provided to the proband. They provided the proband with information about the reason for seeking medical care, which they referred to as a prostate problem requiring specialist care. The word cancer was never used, and the family requested that all clinical staff do the same.

Extensive germline testing revealed a PV in *BRCA2*. The pretest genetic counseling session focused on exploring the family’s intentions regarding partial disclosure. They clarified that the words “cancer” and “chemotherapy” carry a negative meaning and that they would like to provide their father with a message of hope rather than looking at his illness from a negative perspective. This added complexity to the discussion of the results, as the family also decided that his genetic test results and their implications for him and other family members should not be disclosed. The patient was deliberately excluded from the discussion.

Despite the patient’s lack of knowledge about his condition, genetic counseling was successful in involving the younger generation, as more than half of his children consented to cascade testing shortly after the genetic result disclosure, with further appointments scheduled for the remaining siblings.

#### 3.3.8. Case 8

The proband was a 71‐year‐old university‐educated man who was diagnosed with PCa (disease details not recorded) at the age of 65 and later with sigmoid colon adenocarcinoma at the age of 68. He was fully aware of both cancer diagnoses and actively participated in his medical management and in the genetic counseling process.

The patient’s family history showed several malignancies, mostly colorectal cancers. Two of his siblings died from colon cancer in their late 60s, his father was diagnosed with PCa in his late 80s, and his paternal grandmother was reportedly diagnosed with breast cancer in her 50s. There were no cancers in the next generation. Germline analysis identified an LP *BRCA2* variant and several VUSs.

Genetic counseling was successful in discussing hereditary cancer risk with the patient’s immediate family members. One of his daughters, who is in her 30s, underwent cascade testing and started early surveillance. Whether the message of the potential cancer risk was passed on to other relatives is unknown. However, no other relatives had pursued testing at the time of reporting.

#### 3.3.9. Case 9

The proband was a 67‐year‐old working individual with primary‐level education, diagnosed with high‐risk PCa two years ago (initial PSA 10.5 ng/mL, Gleason score 5 + 5 = 10). He showed a good level of understanding of his condition and treatment plan.

The family history is restricted. It consists of a father who died from an unconfirmed bone/soft tissue malignancy of unknown type that was diagnosed at an advanced age. A healthcare‐literate niece supported the proband and attended all his medical appointments.

The proband was fully counseled in the presence of his niece and children. He gave his informed consent for genetic testing. Germline testing revealed a pathogenic *BRCA2* variant. As the patient was approached during his chemotherapy for result disclosure, his niece and children indicated that they would like to discuss his results in his absence. We asked the patient about his preferences, as the information is very important to him, but he said he also preferred that we discuss it with his family members. He was encouraged to contact the genetics team if he had any questions in the future. The result was then disclosed to his niece (who works in a healthcare field) in a separate private consulting room. The niece expressed relief that the *BRCA2* PV result was not disclosed to the proband, as the family feels the proband is currently in “good spirits” and wants him to focus on his chemotherapy now. The plan is to disclose the result to him in due time, as they want to keep him involved, but only when they feel he will be ready to hear it.

Predictive counseling was provided to the at‐risk relatives. The niece and some of the probands’ daughters had cascade testing within a week of the result consultation. His sons, who had participated in pretest counseling and had been informed of the *BRCA2* PV and its associated risk, did not come forward for cascade testing at the time of reporting.

### 3.4. Thematic Findings

A qualitative review of the genetic counseling documentation across the nine cases revealed several recurring sociocultural and communication‐related themes. These themes profoundly influenced the trajectory of genetic counseling and the ultimate uptake of cascade testing. The primary themes identified included varying degrees of family‐mediated protective nondisclosure, the impact of consanguinity and large family structures, barriers related to health literacy and geographic fragmentation, and gendered perceptions of *BRCA2* risk. Table 3 provides a concise mapping of how these key themes manifested within each individual case.

**TABLE 3:** Thematic mapping of sociocultural and communication factors by case.

Case	Family‐mediated protective nondisclosure	Consanguinity/family structure	Health literacy and geographic factors	Gendered risk perception	Cascade testing uptake
1	Yes (proband excluded from genetic results to prevent distress)	Yes (consanguineous marriage, multigenerational cancer)	Standard	Not prominently noted	High
2	No (proband fully informed and involved)	No	Standard	Yes (male relatives viewed *BRCA* as a female issue)	Low
3	Yes (proband kept unaware of both cancer and genetic status)	Yes (consanguineous marriage, multiple inherited risks)	Standard	Not prominently noted	High
4	Partial (proband delayed testing until own second primary diagnosis)	No	Standard	Yes (sons reportedly emotionally unprepared to test)	Moderate
5	Yes (proband unaware of disease progression)	No	Low health literacy; geographically fragmented (migration)	Not prominently noted	Low
6	Yes (historical family nondisclosure regarding relatives’ diagnoses)	Yes (consanguineous marriage)	Limited	Yes (adult sons did not pursue testing)	Low
7	Yes (proband excluded from exact diagnosis and genetic results)	No	Standard	Not prominently noted	High
8	Partial (details of family sharing unknown)	Yes (consanguineous marriage)	Standard	Not prominently noted	Low
9	Yes (results temporarily withheld during chemotherapy)	No	Standard (family member in the medical field)	Yes (sons did not come forward for testing)	Moderate

## 4. Discussion

This case series illustrates the intricate relationship between inherited cancer risk, family structures, and sociocultural factors in the provision of genetic counseling for *BRCA2*‐related PCa in Oman. Although all probands in this case series had P/LP variants in the *BRCA2* gene, the outcomes of genetic counseling and cascade testing differed significantly among them. The differences noted in each case appeared to be influenced less by the severity of the condition or the educational level of the probands and more by the patterns of disclosure, family structures, health literacy, and sociocultural perceptions of illness and inheritance.

### 4.1. Patterns of Disclosure and Family‐Mediated Decision‐Making

One content domain that recurred in several cases (1, 3, 6, 7, and 9) was the partial or total withholding of cancer information or genetic test results from the proband. In such cases, the decision was made jointly by the adult children or spouses, with the aim of shielding the patient from psychological trauma. This finding is in strong agreement with qualitative studies conducted in the region, which identify “intentional withholding” as a protective buffering strategy rather than a deceptive one [17]. In the Omani healthcare context, it is not uncommon for families to serve as surrogates. These family members frequently ask healthcare providers to withhold clinical information to shield their loved ones from psychological trauma [21]. As a result, the amount and timing of the medical information available to the patient are controlled by the family [22]. In addition, words such as “cancer” and “chemotherapy” were avoided in some families because of their negative associations, and the genetic test results were sometimes withheld until a “better time.” This indicates the communication strategies used in the region, where gentler terms such as “tumor” (waram) are preferred first to avoid the shock associated with the word “cancer” (saratan) [21]. It is important to note that the family‐mediated protective nondisclosure observed in this series was not driven by malicious intent but rather by a profound desire to protect vulnerable family members. Recognizing this protective framing allows counselors to work with families from a place of empathy while simultaneously promoting transparency and preventive measures.

In contrast, Western bioethical frameworks have been more focused on the principles of autonomy and full disclosure. Nevertheless, in collectivist cultures such as those in Oman and the broader Middle East, healthcare decisions are often situated within family systems [19]. Indeed, literature indicates that in cultures that value beneficence and nonmaleficence, there is a long history of family‐centered healthcare decision‐making [21]. As has been observed in more recent genetic counseling literature, autonomy in such settings may be understood as relational autonomy, in which patients prefer or rely on family‐centered decision‐making [20]. In our case, genetic counselors repeatedly face a dilemma: balancing family hierarchies and cultural values with their own commitment to transparency and informed decision‐making. Culturally competent care, in this case, means that genetic counselors must be aware of these complex family dynamics rather than simply relying on principles of noninterference or individualism [20].

Notably, nondisclosure did not always prevent cascade testing. In Cases 1, 7, and 9, although the proband was not included in the disclosure, family‐centered counseling helped ensure a high rate of cascade testing in children. In contrast, in Cases 2 and 6, in which patients were fully informed and involved, cascade testing remained low. This suggests that patient autonomy alone may not dictate the rate of cascade testing; rather, communication patterns within families and intergenerational beliefs likely play a significant role. These observations are consistent with the idea of “familial uncertainty management,” whereby risk appraisal and the need for testing are family processes rather than individual decisions [23]. Even if individual autonomy is undermined in probands or if families have a “closed” character concerning medical matters, strong intergenerational relationships and family‐based interventions can successfully encourage family members to undergo preventive testing [16, 23].

Ultimately, although family‐mediated protective nondisclosure is deeply rooted in Omani and broader Middle Eastern collectivist culture [19, 21], recent literature demonstrates that delegating disclosure of genetic results to family members is a recognized clinical phenomenon globally, particularly in oncology. For instance, Murphy et al. [24] observed that even within highly individualistic Western healthcare settings, cancer patients facing poor prognoses frequently exercise their “right not to know” by explicitly requesting that genetic test results be disclosed directly to their children. This parallel indicates that accommodating family‐mediated disclosure in the Omani context is not necessarily a departure from ethical care but rather a culturally adapted application of relational autonomy. Recognizing this allows genetic counselors to bridge the gap between complex, protective family dynamics and the urgent clinical need for cascade testing, ensuring that patients are emotionally supported, whereas at‐risk relatives are still empowered to take preventive action.

### 4.2. Protective Nondisclosure Leading to Missed Opportunities for Early Identification

Some cases (1 and 6, in particular) illustrated how the incomplete communication between the branches of extended families, as well as between different healthcare facilities, contributed to the late assessment of the risk of hereditary cancer. In cases where P/LP variants of the *BRCA2* gene had been previously identified in relatives, this information had not been passed on to the probands or their immediate families. In Case 6, the proband was not aware of his daughter’s breast cancer or her genetic status before his own diagnosis. These communication deficits likely contributed to missed opportunities for cascade testing and surveillance. These clinical observations align with recent regional literature, which suggests that a cultural preference for privacy, withholding, and concealment may hinder extended family members’ access to life‐saving genetic information [16, 17].

In consanguineous families (Cases 1 and 3), the effects of nondisclosure were even more significant. The presence of multiple pathogenic and risk‐associated variants in Case 3 illustrates the potential for amplifying hereditary risk in regions where consanguinity is prevalent. The amplification of risk that has been observed in our cases is supported by regional genomic studies, which have found high levels of consanguinity (25%–60% of marriages) to be the main factor for the high prevalence of hereditary cancer syndromes in Arab populations [19, 25]. In these regions, genetic counseling must extend beyond the standard nuclear family paradigm. Counselors must actively consider complex kinship networks, overlapping inheritance patterns, and the unique dynamics of large extended families.

These cases indicate that genetic counseling in the Omani population, and possibly the broader Middle Eastern region, should consider the potential for incomplete family histories, selective disclosure, and care across multiple centers. This is supported by existing literature, which indicates that genetic counselors in the Middle East often encounter incomplete family histories because some patients are reluctant to provide information about their medical history because of fear of social stigma or “genetic blame” [16].

### 4.3. Gender‐Based Perceptions of *BRCA* Risk

Gender differences in risk perception for *BRCA* were apparent in several cases (2, 4, 6, and 9). Male relatives often felt that the relevance of *BRCA* testing was more for women because of its strong linkage with breast cancer [23]. In Cases 2 and 6, the adult sons did not undergo cascade testing despite counseling and awareness of risk. However, daughters were more likely to undergo testing, as evident in Cases 1, 8, and 9. These clinical findings reflect broader trends in the current literature, which suggest that men are significantly less likely than women to undergo cascade genetic testing and often exhibit higher rates of test refusal and avoidance [10, 14, 26].

This gendered view might reflect larger cultural narratives where inherited breast cancer is seen as a female problem. In addition, female breast cancer and male PCa risk are less talked about in public. Research has shown that the way *BRCA* mutations are framed, commonly referred to as hereditary breast and ovarian cancer (HBOC) syndrome, may contribute to a gendered approach where men tend to underestimate their own risk [11, 13, 27]. Counseling practices in the region might, therefore, require a deliberate focus on male‐specific cancer risks for *BRCA1/2,* such as prostate and pancreatic cancers, to dispel myths. Genetic counseling practices in the region may benefit from being tailored to emphasize male‐specific medical management, rather than the current emphasis on risks to female relatives, to increase male engagement and active health practices [11, 28, 29].

### 4.4. Literacy, Migration, and Fragmented Family Networks

The presence of limited health literacy (Case 5) and geographically dispersed families compounded the challenges of cascade testing. Even without a nondisclosure agreement, the lack of timely and comprehensive communication limited the scope of uptake. These structural issues, alongside nondisclosure, appear to contribute to a lack of comprehensive awareness and poor adherence to preventive measures. These points resonate with regional research highlighting low genetic literacy and geographic fragmentation as potential barriers that may increase the challenges of counseling and hinder clinical compliance [16, 30].

The possible regional clustering of the *BRCA2* c.2588dup variant observed in Cases 4, 5, and 7 also raises further questions about founder effects and the importance of population‐based studies in Oman. This observation is supported by recent genomic analyses, which have independently identified the *BRCA2* c.2588dup variant in Omani populations, thereby emphasizing the region’s unique genetic profile [19]. It is important to note that the literature clearly emphasizes the need to identify local founder mutations in Arab populations, as this would enable the development of highly cost‐effective, population‐specific screening strategies [25, 31].

### 4.5. Implications for Culturally Responsive Genetic Counseling

Taken together, these cases raise the possibility that genetic counseling in this Omani setting may be most effective when it is both client‐centered and culturally sensitive. Client‐centered care does not necessarily mean strict adherence to highly individualistic Western models of autonomy. Important points that have emerged in this series of cases include the following:

1. Relational autonomy: The decision‐making process may properly include representatives of the family; the counselor should determine patient preferences regarding disclosure and involvement rather than assuming a particular model.
2. Correction of gender misconceptions: The counseling session should specifically include male cancer risks associated with *BRCA2.*
3. Early discussion of disclosure plans: Discussion of how and when results will be shared may prevent later ethical conflicts.
4. Follow‐through counseling: Follow‐up sessions may be more effective than single counseling sessions.
5. Considering consanguineous and extended family relationships: Risk communication may require communication beyond the nuclear family.
6. Families are often managed across different institutions, leading to fragmented care. Establishing formalized, consent‐driven information‐sharing pathways between tertiary centers could reduce this fragmentation and prevent missed opportunities for cascade testing. Any such mechanisms must adhere to national data protection regulations, institutional ethical standards, and explicit patient consent processes to safeguard confidentiality and maintain trust.

## 5. Strengths and Limitations

This study offers a highly detailed, real‐world perspective on genetic counseling at a comprehensive cancer center in the Middle East. However, the study is limited by its small sample size, retrospective design, and reliance on recorded genetic counseling sessions. Additionally, there was no long‐term follow‐up of cascade testing outcomes or psychological effects. Although we briefly summarized the initial impact of *BRCA2* status on targeted therapy (e.g., PARP inhibitor eligibility) and high‐risk screening, a comprehensive evaluation of long‐term oncological treatment pathways, medication compliance, and survival outcomes was outside the primary scope of this qualitative genetic counseling review.

## 6. Future Directions and Research Implications

The findings from this case series underscore the need for additional qualitative research to better understand the underlying motivations, beliefs, and communication styles that influence genetic counseling outcomes among Omani families. Although this study identified recurring patterns in family‐mediated protective nondisclosure, gendered perceptions of *BRCA*‐related risk, emotional preparedness, and family‐mediated decision‐making, the retrospective design and reliance on medical records limited deeper exploration of these issues. Future studies using semistructured or qualitative interviews with probands, spouses, adult offspring, and extended family members would provide a greater understanding of how families think about hereditary cancer risk, how disclosure decisions are made, and how cascade testing is understood within cultural, religious, and generational frameworks. These studies might investigate the following questions:

• How do families think about “protection” in the context of nondisclosure?
• How are health decisions allocated within family hierarchies?
• What are the anticipated emotional or social implications of genetic testing?
• How do gender roles affect perceptions of *BRCA*‐related risk?
• How does consanguinity affect understanding of hereditary disease?

Moreover, longitudinal research exploring the dynamics of attitudes toward disclosure and cascade testing over time, especially after the first counseling sessions, could help distinguish between resistance as a sign of unchangeable opposition and delayed readiness, particularly among younger individuals facing educational, professional, and family commitments. This kind of research would greatly contribute to a better understanding of the issues surrounding hereditary cancer disclosure in Oman and the broader Middle Eastern region and help establish counseling frameworks tailored to local culture.

## 7. Conclusion

This single‐center Omani case series illustrates that the effectiveness of genetic counseling in BRCA2‐associated prostate cancer depends not only on achieving a molecular diagnosis to guide treatment but also on the sociocultural context in which counseling is delivered. Our findings demonstrate that family dynamics, communication patterns, cultural beliefs, and prevailing ethical norms can substantially influence the disclosure of genetic information, cascade testing, and access to appropriate surveillance. These observations support the need for culturally responsive genetic counseling models that move beyond the direct application of Western frameworks. This need is further supported by emerging regional evidence demonstrating that standard Western clinical tools, such as the Manchester Scoring System, require adaptation for Arab populations because of factors including consanguinity and large family structures (van Wyk et al., 2025). Collectively, these findings highlight that integrating cultural context into genetic counseling is essential to translating molecular diagnoses into meaningful clinical and preventive outcomes while maintaining rigorous ethical standards.

## Author Contributions

The first and corresponding author, Chantel van Wyk, is the principal investigator. This author conceptualized and executed the study (obtained ethics approval, facilitated data collection, performed data analysis, and wrote the manuscript). The major input to the initial manuscript, pre‐ and postreview, was provided by the second author, Reem Abdulrahim. She updated the clinical treatment and screening information for all cases in the final manuscript. Munjid Al Harthy and Abeer Al Saegh were coinvestigators and assisted in the study’s conceptualization. The last author, Munjid Al Harthy, and Bushra Al Muhairi were part of the data collection team. Chantel van Wyk, Ilse Crous, Reem Abdulrahim, Amira Al Balushi, Bushra Al Muhairi, Sara Al Kiyumi, and Suad Al Malki collected clinical and genetic data by conducting genetic counseling sessions and documenting them in detail.

## Funding

The authors received no financial support for the research, authorship, and/or publication of this article. Open access publishing facilitated by United Arab Emirates University, as part of the Wiley ‐ United Arab Emirates University agreement.

## Disclosure

This work has not been published nor is it under evaluation with any other journal. All authors reviewed and approved the final version of the manuscript and agreed to be accountable for all aspects of the work.

## Ethics Statement

Ethical approval was obtained from the SQCCCRC Institutional Review Board and Ethics Committee (SQCCCRC‐IRB&EC/160/2024/CCCRC‐76‐2024).

## Consent

Written consent was obtained from the participants and their family members involved in consultations for genetic testing and in this research.

## Conflicts of Interest

The authors declare no conflicts of interest.

## Data Availability

The data that support the findings of this study are available on request from the corresponding author. The data are not publicly available due to privacy and ethical constraints.
